# Plasma-Activated Water as a Novel Irrigation Strategy for Seawater-Immersed Burn Wounds: Antibacterial Activity and Healing Promotion in Rats

**DOI:** 10.3390/biomedicines14051027

**Published:** 2026-04-30

**Authors:** Shanshan Wei, Ru Yang, Tian Fang, Zhuo Dai, Xinyu Wang, Yajun Zhao, Sen Wang, Lin Sun

**Affiliations:** 1 Nursing College, Nanjing University of Chinese Medicine, Nanjing 210023, China; weisshan977@njucm.edu.cn (S.W.); 20231015@njucm.edu.cn (R.Y.); 2 Department of Comparative Medicine, Jinling Clinical Medical College, Nanjing University of Chinese Medicine/General Hospital of Eastern Theater Command, Nanjing 210002, China; fangtianlove@126.com; 3 Department of Burns and Plastic Surgery, Jinling Clinical Medical College, Nanjing University of Chinese Medicine/General Hospital of Eastern Theater Command, Nanjing 210002, China; daizhuo237117002@163.com; 4 Surgical Department of Emergency Medicine, War Trauma Treatment Center, Jinling Clinical Medical College, Nanjing University of Chinese Medicine/General Hospital of Eastern Theater Command, Nanjing 210002, China; 18851190488@163.com; 5 Electrical Engineering and Control Science College, Nanjing Tech University, Nanjing 211816, China; zhaoyajun@njtech.edu.cn (Y.Z.); wang_sen@njtech.edu.cn (S.W.); 6 Department of Military Casualty Management, Jinling Clinical Medical College, Nanjing University of Chinese Medicine/General Hospital of Eastern Theater Command, Nanjing 210002, China

**Keywords:** plasma-activated water, seawater-immersed burn wound, wound infection management, wound healing, wound irrigation

## Abstract

**Objectives:** Seawater-immersed burn wounds are highly susceptible to contamination, persistent inflammation, oxidative stress, and delayed healing, while current irrigation solutions remain suboptimal for such acute injuries. This study aimed to evaluate the therapeutic efficacy and underlying mechanisms of plasma-activated water (PAW) as a novel irrigation strategy for these complex wounds. **Methods:** The antibacterial efficacy of PAW against marine pathogens was first evaluated in vitro. Subsequently, a rat model of seawater-immersed burn injury was established in male Sprague-Dawley (SD) rats to assess the therapeutic effects of PAW irrigation on wound healing, infection control, and underlying biological mechanisms. **Results:** In vitro, PAW significantly eradicated two major marine pathogens, *Vibrio vulnificus* and *Vibrio parahaemolyticus* (*p* < 0.001). In vivo, PAW markedly accelerated wound closure, achieving complete healing in 23.60 ± 6.50 days vs. 38.67 ± 2.08 days (Normal saline group) and 58.33 ± 10.97 days (Model group) (*p* < 0.05). PAW significantly reduced bacterial burden, modulated inflammation by decreasing interleukin-6 and increasing interleukin-10, and alleviated oxidative stress, as evidenced by reduced malondialdehyde levels and enhanced superoxide dismutase activity. Histological evaluation demonstrated enhanced re-epithelialization, collagen deposition, and increased expression of vascular endothelial growth factor and platelet endothelial cell adhesion molecule-1. No adverse effects on serum biochemistry or major organ histopathology were observed. **Conclusions:** PAW may be a safe, promising, and multifunctional irrigation strategy that promotes seawater-immersed burn healing through coordinated antibacterial, anti-inflammatory, antioxidant, and pro-angiogenic effects, highlighting its strong potential for clinical translation.

## 1. Introduction

Seawater-immersed burns are emerging as an increasingly serious global health concern. The expansion of maritime industries has increased the frequency of offshore operations, including commercial shipping, exploration, and fishing. Individuals working in maritime and port operations involving high-energy systems or volatile cargo—on vessels, offshore platforms, and in on-shore facilities such as loading docks and warehouses—are exposed to an increased risk of explosions and fires, which contributes to a higher incidence of burn injuries [1]. Compared to simple burn injuries, immersion in seawater following maritime accidents complicates burn wounds due to the hypertonic, alkaline, low-temperature, and inorganic-salt-rich environment. For instance, hypertonicity induces localized edema, vascular leakage, and intracellular dehydration, thereby compounding the initial thermal injury [2]. Furthermore, seawater immersion is associated with a higher infection rate and significantly elevated mortality due to higher bacterial loads [3,4]. Seawater bacteria, primarily from the Vibrio genus, can invade broken skin and significantly impede wound healing. The annual increase in the incidence of Vibrio infections has drawn considerable attention, and pathogens such as *Vibrio vulnificus* and *Vibrio parahaemolyticus* have posed a serious threat, causing wound infections, necrotizing fasciitis, and systemic complications upon ingestion [5,6]. Without timely care, such dirty wounds may lead to persistent inflammation and systemic oxidative stress [7,8]. Ultimately, these factors may impede the healing process and increase the likelihood of chronic wound problems, then contribute to elevated morbidity and mortality [9].

Early and comprehensive irrigation is the first step in wound debridement [10]. It can remove contaminants, necrotic tissue, and bacterial bioburden, ultimately reducing infection risk and creating a wet-healing microenvironment [11,12]. Standard irrigation fluids include normal saline, sterile water, povidone-iodine, and hydrogen peroxide [13,14]. Among these, normal saline is the most frequently used irrigation solution; however, it cannot kill the pathogens by itself, as sterile water does [15]. Solutions such as povidone-iodine, hydrogen peroxide, and antibiotics have bactericidal properties; however, they may cause toxic side effects, such as cytotoxicity in fibroblasts and keratinocytes, delayed epithelial regeneration, tissue irritation, and allergic reactions, which can slow healing and lead to bacterial resistance [16,17,18,19]. In general, existing irrigation solutions are not effective in treating complex wounds, and there is ongoing debate about their effectiveness against specific marine microorganisms and against combined wounds in seawater [1,20,21]. Therefore, identifying an optimal irrigation solution that combines strong antibacterial activity, low toxicity, and wound healing promotion is a key aspect of wound care.

Recently, plasma technology, an emerging multifunctional physicochemical material with broad application prospects, has attracted significant interest in wound management. Biomedical applications of plasma encompass direct treatment, in which ionized gas physically contacts target tissues, and indirect treatment, which uses plasma-activated media (such as activated water, oils, or hydrogels) to exert therapeutic effects [22]. In particular, plasma-activated water (PAW) is a novel functional solution generated by treating solutions, such as sterile water or normal saline, with cold atmospheric plasma (CAP), and it is rich in various active substances, primarily reactive oxygen species (ROS) and reactive nitrogen species (RNS) [23]. Numerous research findings have demonstrated that PAW can inactivate various bacterial strains, modulate inflammatory responses, and significantly promote wound healing [24,25,26]. The mechanism of PAW in wound closure involves restoring the dynamic balance of the inflammatory microenvironment, stimulating the migration and proliferation of fibroblasts and keratinocytes, and inducing angiogenesis to accelerate tissue remodeling [24,27]. Because of its unique physicochemical properties and excellent biocompatibility, PAW is hypothesized to be an ideal irrigation solution for seawater-immersed burn wounds.

This study aims to examine the antibacterial efficacy of PAW using in vitro assays and to assess its effectiveness on seawater-immersed burn wounds in rats, thereby providing experimental proof and a theoretical foundation for clinical therapy.

## 2. Materials and Methods

### 2.1. Preparation of PAW

**Figure 1:**
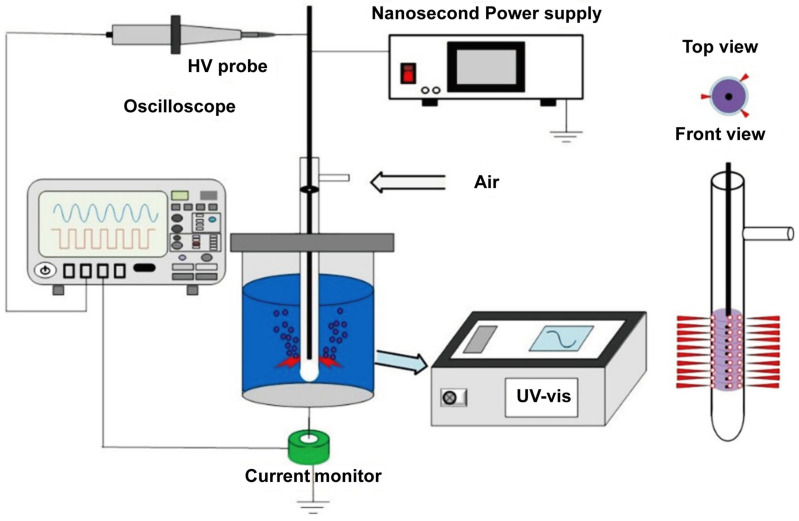
Schematic diagram of the PAW preparation system and its working principle. The system is driven by a nanosecond pulsed power supply. Air is introduced as the working gas into the bubble discharge reactor. The plasma generated inside the quartz tube spreads out through the sidewall holes to interact with 100 mL of normal saline (Note: The flexible metal net serving as the ground electrode around the quartz container is not plotted to better visualize the internal discharge pattern).

The plasma generator independently developed by our team was adopted. The PAW generation system consisted of a nanosecond pulsed power supply, a bubble discharge reactor, and a diagnosis system. A nanosecond pulse power supply was used to generate pulsed voltages up to 7 kV and 1 kHz, with the pulse rising and falling time of 50 ns, and the pulse width of 1000 ns. The bubble discharge reactor consisted of a quartz tube (height: 170 mm, thickness: 1 mm). On the sidewall, ten rows of holes were drilled, each with a diameter of 1 mm. In each row, three holes were spaced 120° apart. A high-voltage electrode made of a 5 mm diameter iron rod was placed coaxially inside the quartz tube. The bottom end of the electrode was aligned with the lowest row of holes. A quartz container was employed to hold 100 mL normal saline, with a flexible metal net wrapped around the outside wall of the container as the ground electrode. In the figure, to better visualize the discharge pattern inside, the ground electrode was not plotted. Air was introduced into the reactor through a branch pipe inlet as the working gas. When the pulsed voltage was applied on the high-voltage electrode, the discharge occurred between the electrode and the quartz tube, then plasma spread out of the quartz tube and interacted with the normal saline to generate aqueous species. The solution was treated for 10 min to prepare PAW and then set aside for subsequent experiments (Figure 1). The concentrations of the three types of long-lived active particles, nitrate (NO_3_^−^), hydrogen peroxide (H_2_O_2_), and nitrite (NO_2_^−^) were measured using an ultraviolet spectrophotometer(UV-1800, Shanghai Meipuda Instrument Corp., Shanghai, China). In addition, the pH value and oxidation–reduction potential (ORP) of PAW were measured using a calibrated pH meter (PHS-3E, Leici Instrument Corp., Shanghai, China), while the electrical conductivity (EC) was measured using a conductivity meter (DSS-11A, Leici Instrument Corp., Shanghai, China). All measurements were performed immediately after PAW preparation, with each sample measured in triplicate.

### 2.2. Antibacterial Effectiveness In Vitro

#### 2.2.1. Bacterial Suspension Preparation

*Vibrio vulnificus* (ATCC 27562) and Vibrio parahaemolyticus (ATCC 17802) lyophilized cultures were obtained from the Guangdong Institute of Microbiology (Guangzhou, Guangdong, China). The strains were taken out, dripped with 3% NaCl alkaline peptone water, applied to 2216E agar plates (Hopebio Corp., Qingdao, Shandong, China) in 90 mm sterile Petri dishes (BKMAMLAB Corp., Changde, Hunan, China), cultured at 37 °C for 18–24 h, successfully resuscitated, and plate lineage, and cultured again at 37 °C for 18–24 h. Sterile pick-and-place single colonies were inoculated into CAMHB liquid medium containing 3% NaCl (Hopebio Corp., Qingdao, Shandong, China), placed on a 37 °C, 180 rpm shaker, shaken for 18–24 h, and adjusted to approximately 1 × 10^8^ CFU/mL.

#### 2.2.2. Viable Bacterial Count Experiment

Each bacterial suspension was diluted to 2 mL and adjusted to about 10^6^ CFU/mL, then transferred to 5 mL centrifuge tubes and mixed with PAW at a 1:1 ratio, with normal saline as the control. Then, 100 μL was taken at 5 min and subjected to serial tenfold dilutions, and 100 μL of the diluted bacterial suspension was uniformly spread onto 2216E agar plates in 90 mm sterile Petri dishes and cultured at 37 °C for 24 h. Each experiment was replicated three times, and the number of live bacteria per plate was manually counted using the Multi-point tool in ImageJ and recorded.

### 2.3. Wound Healing In Vivo

#### 2.3.1. Animal Preparation

All 48 male Sprague-Dawley (SD) rats (200–220 g, 6–8 weeks old, SPF grade) were used to ensure baseline consistency. Rats were housed under standard conditions with free access to food and water and acclimatized for one week before experimentation. All study methods and procedures met requirements from the Animal Research: Reporting of In Vivo Experiments (ARRIVE2.0) guidelines [28].

#### 2.3.2. Sample Size Calculation

The sample size was calculated using the Resource Equation Determination Method, based on the error degrees of freedom (E) in the analysis of variance model [29]. To ensure statistical validity, E was recommended to range from 10 to 20 [29]. The formula was as follows. Calculations indicated that 4–6 rats per group were sufficient to achieve statistical significance. Considering the need for batch sampling on days 7 and 14 during the experiment, a total of 48 rats were projected to be used.

(1) $$E=N-K=Kn-K=K\left(n-1\right)$$

(2) n=E/K+1

The N meant the total number of experimental units, the K meant the number of groups, and the n meant the sample size per group.

#### 2.3.3. Preparation of Seawater Solution

The experimental seawater solution was prepared in accordance with the primary constituents of seawater from the southeastern coast of our country, as furnished by the Marine Chemistry Research Office of the Third Laboratory of the State Oceanic Administration: NaCl (Sinopharm Corp., Shanghai, China) 26.52 g/L, MgSO_4_ (Sinopharm Corp., Shanghai, China) 3.31 g/L, MgCl_2_ (Sinopharm Corp., Shanghai, China) 2.447 g/L, CaCl_2_ (Sinopharm Corp., Shanghai, China) 1.14 g/L, KCl (Kermel Corp., Tianjin, China) 0.73 g/L, NaHCO_3_ (Kermel Corp., Tianjin, China) 0.20 g/L, NaBr (Fuchen Corp., Tianjin, China) 0.08 g/L, osmotic pressure 1300 mmol/L, pH 8.2, solution temperature (22 ± 1) °C, laboratory room temperature 25 °C. These reagents were dissolved in ultrapure water (Elkay Corp., Downers Grove, IL, USA). The prepared solution was sterilized via high-pressure steam autoclaving (Zhiwei Corp., Xiamen, Fujian, China), was not filtered, and was left to stand for subsequent use. A fresh mixed bacterial suspension was added to the seawater solution, and the concentration was calibrated to 10^7^ CFU/mL before use.

#### 2.3.4. Establishment of Animal Models

**Figure 2:**
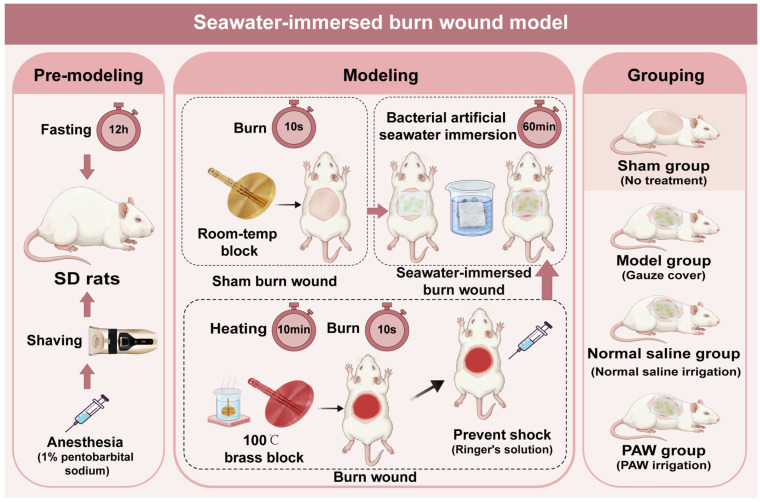
Schematic diagram of the establishment and treatment grouping of the seawater-immersed burn wound model in SD rats. The figure illustrates the standard procedures of pre-modeling, modeling (burn induction and bacterial seawater immersion), and the final allocation into four specific treatment groups (Sham, Model, Normal saline, and PAW). Created in BioRender. Liu Tian. (2026). https://BioRender.com/xlvqblv (accessed on 26 April 2026).

Fasting was performed for 12 h before injury. A solution of 1% pentobarbital sodium (40 mg/kg, Biopike Corp., San Jose, CA, USA) was used for anesthesia. In total, 30% of the back area was prepared, the prone position was maintained, and a 5.0 cm × 5.0 cm burn area was marked on the back. A 90 g brass block with a diameter of 5 cm was immersed under the water reservoir maintained at a constant setting of 100 °C and subjected to heating for a duration of 10 min, taken out and dried, and placed in the middle of the back of the rats in the experimental group for 10 s to induce a second-degree burn injury. Ringer’s solution 2 mL was immediately administered intraperitoneally to prevent shock. Subsequently, sterile gauze was completely immersed in the prepared bacteria-containing artificial seawater and then used to fully cover the entire burn wound, ensuring continuous, uniform contact between the wound surface and the seawater. During the 60 min exposure period, the gauze was kept fully saturated by periodically adding the same artificial seawater to maintain a consistently moist environment. Then, the remaining seawater on the wound surface was dried with sterile gauze blocks, and a seawater-immersed burn wound model was established. The sham group served as the control, with the same procedure performed using brass blocks maintained at room temperature (Figure 2). To avoid confounding the assessment of target inflammatory markers (IL-6 and IL-10) and the natural progression of wound healing, no post-operative analgesics or anti-inflammatory drugs were administered during the routine dressing changes.

#### 2.3.5. Groups and Treatments

Randomization was performed after successful model establishment, using a random sequence generated in IBM SPSS Statistics version 27.0. Animals assigned to the same experimental group were housed in the same cage to avoid cross-intervention interference.

The subsequent treatments were all conducted by the same investigator: (1) Sham group (*n* = 8): no treatment was administered. (2) Model group (*n* = 14): no treatment was administered, and the wound was covered with sterile gauze blocks. (3) Normal saline group (*n* = 13): the wound was disinfected with iodophor, and was then flushed with a 50 mL syringe under maximum manual pressure, while simultaneously being wiped with sterile cotton wool pads saturated in 0.9% normal saline for 5 min. Finally, the wound was covered with sterile gauze blocks. (4) PAW group (*n* = 13): the wound was disinfected with iodophor, and was then flushed with a 50 mL syringe under maximum manual pressure, while simultaneously being wiped with sterile cotton wool pads saturated in PAW for 5 min. Finally, the wound was covered with sterile gauze blocks. The 5 min irrigation duration was determined based on our preliminary in vitro antibacterial results. All treatments were daily for the first week, then on every other day, to manage early exudation while subsequently reducing mechanical damage to the healing wound bed.

#### 2.3.6. Wound Healing Measurement

The wound photographs were collected on days 0, 1, 7, 14, and 21. Wound healing time was marked as the time point at which complete wound closure was observed with the naked eye by the same consistent investigator. The wound areas were manually traced and calculated using the selections tool and Measure function in ImageJ, and the repair ratio was determined as follows:

(3) $$H=\left(S_{0}-S_{X}\right)S_{0}*100\%$$

The S_X_ meant the wound area upon day X, and S_0_ meant the wound area upon day 0.

#### 2.3.7. Bacterial Count

Wound exudate was obtained using sterile, wet cotton swabs on days 1, 3, and 5. After gentle removal of surface debris, wound exudate was obtained using sterile cotton swabs pre-moistened with sterile normal saline. Each swab was applied to the entire wound surface using a standardized “Z”-shaped motion while maintaining consistent moderate pressure to ensure uniform sampling across animals. To minimize variability, the same investigator performed all sampling procedures following the same technique and duration. Immediately after collection, each swab was placed into a sterile centrifuge tube containing 2 mL of normal saline and vortexed thoroughly to elute the bacteria. The resulting bacterial suspension was tenfold serially diluted, and 100 μL of each dilution was spread onto 3% sodium chloride soybean–casein digest agar plates (Hopebio Corp., Qingdao, Shandong, China) in 90 mm sterile Petri dishes. Each experiment was replicated three times, and the number of CFUs was manually quantified using the Multi-point tool in ImageJ and recorded.

#### 2.3.8. Inflammatory Factors Analysis

An enzyme-linked immunosorbent assay (ELISA) was used to detect inflammation-related markers in arterial blood samples. Blood samples were obtained from 4 rats in each group on days 7 and 14 following anesthesia. After standing at room temperature for 2 h, the samples were centrifuged at 3000 rpm for 15 min to obtain the serum. Serum levels of interleukin-6 (IL-6) and interleukin-10 (IL-10) were evaluated strictly according to the manufacturer’s instructions using Rat IL-6 and IL-10 ELISA kits (Thermo Fisher Scientific Corp., Waltham, MA, USA). The absorbance (OD) of the samples was measured at 450 nm using a microplate reader (BioTek Corp., Winooski, VT, USA), and sample concentrations were calculated from the standard curves.

#### 2.3.9. Oxidative-Stress Analysis

On days 7 and 14, 4 rats from each group were euthanized, and wound tissue was collected for analysis of protein content, malondialdehyde (MDA), and superoxide dismutase (SOD) using colorimetric biochemical assay kits. Total protein concentration was determined utilizing a BCA protein assay kit (Servicebio Corp., Wuhan, Hubei, China). MDA content was measured utilizing an MDA assay kit (Servicebio Corp., Wuhan, Hubei, China) via the thiobarbituric acid (TBA) method. SOD activity was measured utilizing a T-SOD assay kit (Servicebio Corp., Wuhan, Hubei, China) via the xanthine oxidase (hydroxylamine) method. All procedures were performed according to the manufacturer’s protocols. Absorbance was recorded using a microplate reader (BioTek Corp., Winooski, VT, USA) at 532 nm for MDA and 450 nm for SOD.

#### 2.3.10. Histological Evaluation

On days 7 and 14, 4 rats from each group were euthanized, and the wound tissue was removed. After fixation with 4% paraformaldehyde, they were stained with Hematoxylin and Eosin (H&E, Servicebio Corp., Wuhan, Hubei, China), and Masson’s trichrome (Masson, Servicebio Corp., Wuhan, Hubei, China) for assessing structure and collagen deposition, and immunohistochemistry staining was used to measure the release of platelet endothelial cell adhesion molecule-1 (CD31) as well as vascular endothelial growth factor (VEGF), utilizing Anti-CD31 Rabbit pAb (Servicebio Corp., Wuhan, Hubei, China), Recombinant Anti-VEGFA Rabbit mAb (Servicebio Corp., Wuhan, Hubei, China), HRP-conjugated Goat Anti-Rabbit IgG (Servicebio Corp., Wuhan, Hubei, China), and a DAB color development kit (Auragene Corp., Changsha, Hunan, China). Stained slides were observed and imaged using an upright optical microscope (DM2700, Leica Corp., Wetzlar, Hesse, Germany). Inflammatory infiltration, collagen deposition, and vascular remodeling during wound healing were assessed through semi-quantitative analysis. The inflammatory cell infiltration area was quantified by calculating the mean relative infiltration area in three to five random 40× fields per H&E-stained sample using the Color Deconvolution 1.7 plugin, followed by the Threshold and Measure functions in ImageJ. Collagen volume fraction (CVF) was quantified as the percentage of the area occupied by collagen deposition in 10× fields per Masson-stained sample using the same Color Deconvolution 1.7 plugin, Threshold, and Measure functions. Microvessel density (MVD) was quantified by counting CD31-positive vessels in three to five random 20× fields per sample using the Multi-point tool in ImageJ. VEGF expression was evaluated at 40× magnification and assigned a Histochemistry-score (H-score), which combines staining grade (0–3+) with the ratio of positively stained cells to yield a value of 0–300. The H-score was automatically calculated using the AIpathwell IHC module in Saiviewer.

#### 2.3.11. Biosafety Analysis

After wound healing, the remaining rats were anesthetized, and serum samples were collected to evaluate blood biochemical indicators using a fully automatic biochemical analyzer (Rayto Corp., Shenzhen, Guangdong, China) to assess systemic hepatic and renal functions, as well as metabolic homeostasis [24]. The specific indicators included liver function markers (albumin [ALB, Rayto Corp., Shenzhen, Guangdong, China], aspartate aminotransferase [AST, Rayto Corp., Shenzhen, Guangdong, China]), a renal function marker (urea nitrogen [BUN, Rayto Corp., Shenzhen, Guangdong, China]), and systemic metabolic/electrolyte indicators (total cholesterol [T-CHO, Rayto Corp., Shenzhen, Guangdong, China], glucose [GLU, Rayto Corp., Shenzhen, Guangdong, China], potassium [K^+^, Getein Biotech Corp., Nanjing, Jiangsu, China]). Then the rats were euthanized, and the internal organs were taken out, including heart, liver, spleen, lungs, and kidneys, for H&E staining.

### 2.4. Quality Control

To minimize bias, specific quality-control and partial-blinding measures were implemented. Macroscopic wound evaluation was performed unblinded, but standardized parameters, including the use of the same camera device at a fixed angle and distance, alongside a standard 5 cm-diameter white reference ring, were applied to ensure strict scale accuracy. Laboratory biological assays of blood and skin tissue samples were conducted by independent technicians blinded to the experimental groups. Histological evaluations were performed unblinded, employing a combination of software-assisted and manual techniques to ensure consistency. Specifically, inflammatory infiltration and CVF were quantified using ImageJ plugins, and VEGF expression was automatically calculated using an AI module, whereas MVD was manually counted. The final statistical data analysis was performed without blinding.

### 2.5. Statistical Analysis

The image data were analyzed using the Fiji distribution of ImageJ (version 1.54p; National Institutes of Health, Bethesda, MD, USA) and the AIpathwell module of Saiviewer (version 2.3.0; Servicebio Corp., Wuhan, Hubei, China). The experimental data were analyzed using IBM SPSS Statistics version 27.0 (IBM Corp., Armonk, NY, USA) and plotted in OriginPro 2025b SR1 (OriginLab Corp., Northampton, MA, USA). The results were presented as “mean ± standard deviation ($\bar{x}$ ± s)”, and the normality of distribution and homogeneity of variance were assessed. For data that conform to a normal distribution and are homoscedastic, independent-samples *t*-tests were employed to compare two groups. One-way analysis of variance (ANOVA) was used for comparisons involving multiple groups, while two-way ANOVA was used for comparisons involving two factors. The Kruskal–Wallis H test or Mann–Whitney U test was used when the data failed to fulfill the normality assumption. For repeated-measures data collected from the same subjects at different time points, a repeated-measures ANOVA or a Friedman test was applied. For all multi-group analyses, including one-way ANOVA, Kruskal–Wallis H, and Friedman tests, post hoc multiple comparisons were adjusted using the Bonferroni method. For two-way and repeated-measures ANOVAs, if a significant interaction effect was detected, pairwise comparisons with Bonferroni correction were performed to assess group differences at individual time points and temporal changes within each group. If the interaction was not significant, Bonferroni post hoc comparisons were conducted for the significant main effects. All *p*-values reported in the text and figures represent these correctly adjusted values. All statistical analyses used a corrected *p* < 0.05 to determine statistical significance.

## 3. Results

### 3.1. Characterization of PAW

**Figure 3:**
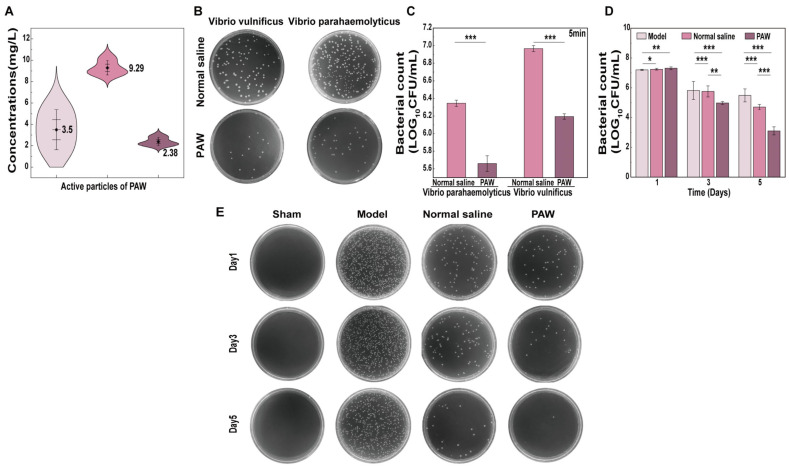
Characterization of plasma-activated water (PAW) and its antibacterial effects in vitro and in vivo. (**A**) The active particle concentrations of PAW (*n* = 6). (**B**) Plates used to culture Vibrio vulnificus and Vibrio parahaemolyticus colonies after PAW treatment (*n* = 3). (**C**) Viable bacterial counts after PAW treatment in vitro (*n* = 3). (**D**) Viable bacterial counts on days 1, 3, and 5 among groups in vivo (*n* = 3). (**E**) Plates of bacterial colonies on days 1, 3, and 5 among groups in vivo (*n* = 3). * marked *p* < 0.05; ** marked *p* < 0.01; *** marked *p* < 0.001.

This study first calibrated the concentration changes in the active components NO_3_^−^, H_2_O_2_, and NO_2_^−^ in PAW under different conditions, and then conducted experiments with fixed, uniform discharge parameters. It was found that the concentrations of NO_3_^−^, H_2_O_2_, and NO_2_^−^ were 11.02 ± 1.91, 9.30 ± 0.67, and 2.38 ± 0.37 mg/L (Figure 3A). The pH, ORP, and EC of PAW were 3.24 ± 0.03, 175.7 ± 1.2 mV, and 15.13 ± 0.02 mS/cm, respectively, while the corresponding values of the control group were 4.58, 98 mV, and 14.87 mS/cm (Table 1).

### 3.2. Antibacterial Effect Analysis

This study examined the antibacterial activity of PAW against the *Vibrio vulnificus* and *Vibrio parahaemolyticus*. It was demonstrated that, in comparison to normal saline, PAW significantly reduced the viable bacterial counts after 5 min of contact with the two suspensions (*p* < 0.001) (Figure 3B,C). Figure 3E shows the quantitative bacterial culture results of wound exudate on days 1, 3, and 5. On day 1, the colony count in the Model group was significantly higher than in the Normal saline and PAW groups (*p* < 0.05). From day 3 onwards, significant differences in colony counts emerged among the three groups (*p* < 0.01) as follows: PAW group < Normal saline group < Model group. On day 5, the PAW group had successfully maintained bacterial counts at extremely low levels; although the Normal saline group was lower than the Model group, it remained significantly elevated compared to PAW group (*p* < 0.001) (Figure 3D).

### 3.3. Promoting the Healing Effect Analysis

#### 3.3.1. Wound Healing Overview

**Figure 4:**
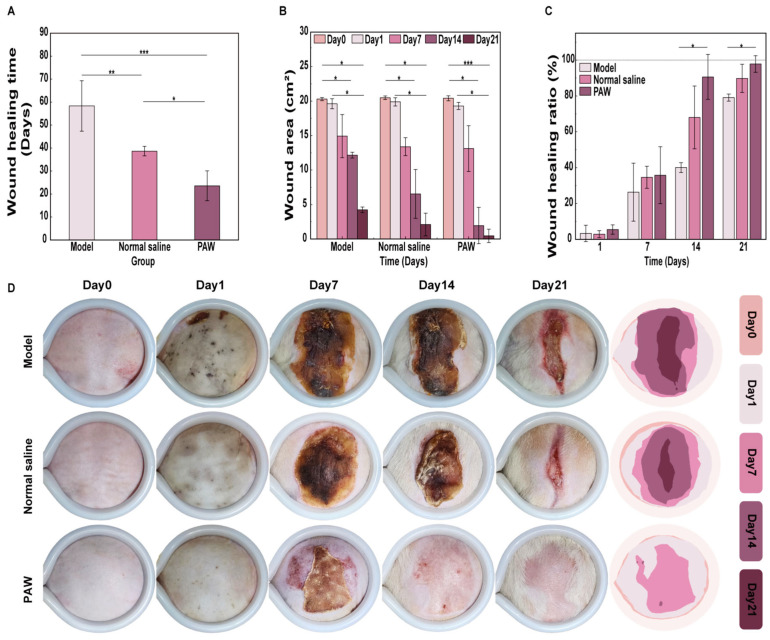
Evaluation of wound healing progression among groups. (**A**) Wound healing times among groups (*n* = 3–5). (**B**) Wound area on days 0, 1, 7, 14, and 21 among groups (*n* = 3–5). (**C**) Wound healing rates on days 0, 1, 7, 14, and 21 among groups (*n* = 3–5). (**D**) Photographs of wounds among groups (*n* = 3–5). * marked *p* < 0.05; ** marked *p* < 0.01; *** marked *p* < 0.001.

Throughout the 21 days, the PAW group consistently demonstrated superior healing speed and quality compared to both other groups. On day 7, scab detachment was faster in the PAW group, with dry, uniform scabs. On day 14, the Model and Normal saline groups still showed significant unhealed areas with partial scab retention, while scab detachment and epithelialization in the PAW group were largely complete. On day 21, the PAW group had achieved almost complete wound closure, whereas residual unhealed areas persisted in the control groups (Figure 4D).

#### 3.3.2. Wound Healing Time

During the experiment, there were three deaths in the Model group (one due to anesthesia) and two in the Normal saline group (one due to anesthesia), while all rats in the Sham and PAW groups survived. The PAW group had a significantly shorter average healing time, achieving complete healing in 23.60 ± 6.50 days vs. 38.67 ± 2.08 days (Normal saline group) and 58.33 ± 10.97 days (Model group). Inter-group comparisons revealed significant differences in healing time (*p* < 0.05), with an extremely large difference observed between the PAW and Model groups (*p* < 0.001) (Figure 4A). This indicated that PAW significantly accelerated the complete wound healing process.

#### 3.3.3. Wound Healing Area

Wound area measurements revealed that, although wound area reduction in the PAW group exhibited non-statistically significant differences at certain early time points (for example, day 1 compared to day 14, uncorrected *p* = 0.046, corrected *p* = 0.455), from day 14 onwards, however, all groups exhibited a significant reduction compared to day 0 (*p* < 0.05), with PAW group highlighting an extremely significant difference (*p* < 0.001) (Figure 4B). Wound-healing ratio measurements revealed non-significant differences among the groups during the early phase (days 1 and 7). However, at the critical time points of days 14 and 21, compared with the Model group, the healing rate of the PAW group was noticeably greater (*p* < 0.05); Notably, compared with the Normal saline group, although the difference did not reach statistical significance, the overall healing rate curve of the PAW group exhibited a continuous upward trend and remained at a consistently higher level, indicating a stable tendency toward improvement (Figure 4C).

### 3.4. Histological Analysis

#### 3.4.1. H&E and Masson Staining Analysis

**Figure 5:**
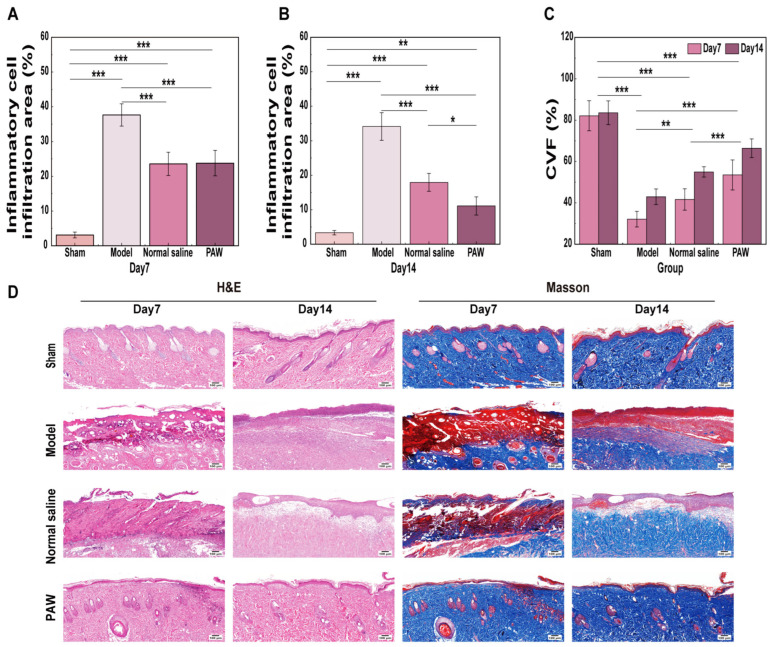
Evaluation of histological inflammation and collagen deposition among groups. (**A**) Inflammatory cell infiltration area on day 7 among groups (*n* = 4). (**B**) Inflammatory cell infiltration area on day 14 among groups (*n* = 4). (**C**) CVF on days 7 and 14 among groups (*n* = 4). (**D**) H&E and Masson staining on days 7 and 14 among groups (*n* = 4) (×100). * marked *p* < 0.05; ** marked *p* < 0.01; *** marked *p* < 0.001.

H&E staining revealed more accelerated, organized tissue repair in the PAW group on day 7 compared with the control groups. This was characterized by accelerated clearance of necrotic tissue, markedly reduced inflammatory cell infiltration, and increased numbers of new capillaries and fibroblasts. On day 14, the inflammatory response in the PAW group had largely subsided, and the granulation tissue had become more mature. Masson staining showed more abundant, denser collagen fiber deposition in the PAW group on day 7 than in the control groups, with a higher blue staining intensity. The collagen fibers in the PAW group were arranged more densely and in a more organized manner, with the collagen deposition morphology approaching that of normal skin on day 14. In contrast, the control groups predominantly demonstrated delayed epithelial regeneration, persistent inflammation, tissue edema, and sluggish collagen synthesis, with sparse, disorganized collagen throughout the observation period (Figure 5D).

The results for the inflammatory cell infiltration area demonstrated a strong association between time and group (*F* = 7.184, *p* = 0.001). On day 7, the inflammatory cell infiltration area in the Model group was greatly higher than in the other groups (*p* < 0.001) (Figure 5A). On day 14, the inflammatory cell infiltration area in PAW group was significantly less than Model and Normal saline groups (*p* < 0.05) (Figure 5B). Additionally, the PAW and Normal saline groups exhibited a significant reduction from day 7 to day 14 (*p* < 0.01). The results for CVF revealed significant main effects for group (*F* = 112.034, *p* < 0.001) and time (*F* = 27.879, *p* < 0.001). All three experimental groups exhibited a significant rise from day 7 to day 14 (*p* < 0.01), and significant differences in CVF among the three groups (*p* < 0.01) as follows: PAW group > Normal saline group > Model group (Figure 5C).

#### 3.4.2. Immunohistochemical Staining Analysis

**Figure 6:**
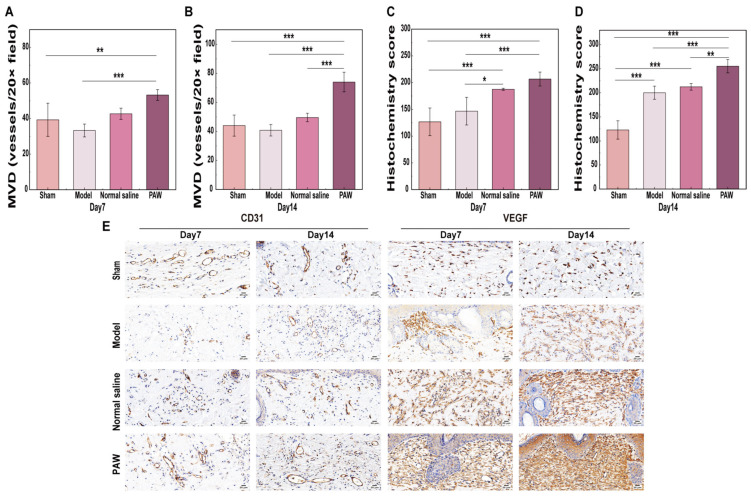
Effects of PAW on angiogenesis and angiogenesis-related factors among groups. (**A**) MVD counts on day 7 among groups (*n* = 4). (**B**) MVD counts on day 14 among groups (*n* = 4). (**C**) H-score of VEGF on day 7 among groups (*n* = 4). (**D**) H-score of VEGF on day 14 among groups (*n* = 4). (**E**) Immunohistochemical staining of CD31 and VEGF on days 7 and 14 among groups (*n* = 4) (×400). * marked *p* < 0.05; ** marked *p* < 0.01; *** marked *p* < 0.001.

PAW treatment significantly increased VEGF and CD31 protein levels in rat wound tissue (Figure 6E). The results for MVD demonstrated a significant interaction between time and group (*F* = 3.51, *p* = 0.031). On day 7, the MVD in the PAW group was significantly greater than in the Sham (*p* < 0.05) and Model (*p* = 0.001) groups (Figure 6A). On day 14, the MVD in PAW group was greater than in the other groups (*p* < 0.001) (Figure 6B). For the H-score of VEGF, a significant time-by-group interaction effect was also demonstrated (*F*= 4.788, *p* = 0.009). On day 7, the PAW and Normal saline groups showed significantly higher levels than the Sham (*p* < 0.001) and Model (*p* < 0.05) groups (Figure 6C). On day 14, all three experimental groups demonstrated significantly greater responses than the sham group (*p* < 0.001), with the PAW group exceeding both other groups (*p* < 0.01) (Figure 6D). All three experimental groups exhibited a significant increase from day 7 to day 14 (*p* < 0.05). In summary, PAW treatment demonstrated the most pronounced effect in promoting MVD and VEGF expression compared to the control group, with its pro-angiogenic effects intensifying over time.

### 3.5. Inflammatory Responses Analysis

On days 7 and 14, this study examined alterations in serum concentrations to assess how the inflammatory resolution phase transitioned into the start of the regenerative repair process. The results demonstrated a significant interaction between time and group for IL-6 (*F* = 7.451, *p* < 0.001) and IL-10 (*F* = 122.288, *p* < 0.001).

**Figure 7:**
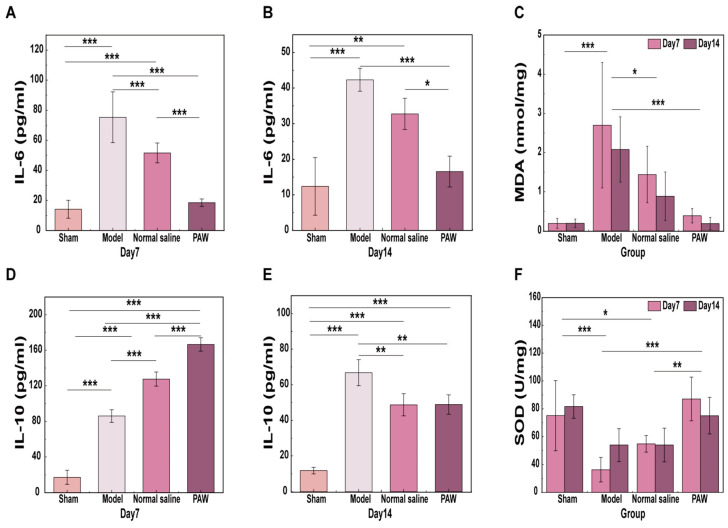
Effects of PAW on cytokine-mediated inflammation and oxidative stress among groups. (**A**) IL-6 levels on day 7 among groups (*n* = 4). (**B**) IL-6 levels on day 14 among groups (*n* = 4). (**C**) MDA levels on days 7 and 14 among groups (*n* = 4). (**D**) IL-10 levels on day 7 among groups (*n* = 4). (**E**) IL-10 levels on day 14 among groups (*n* = 4). (**F**) SOD levels on days 7 and 14 among groups (*n* = 4). * marked *p* < 0.05; ** marked *p* < 0.01; *** marked *p* < 0.001.

On day 7, there were significant differences among the four groups for IL-6 (*p* < 0.001). Notably, the PAW group was significantly lower than both other experimental groups (*p* < 0.001), while no significant difference was observed between the PAW group and the sham group (Figure 7A). On day 14, the IL-6 levels in PAW group were still significantly less compared to both experimental groups (*p* < 0.05) (Figure 7B). For IL-10, significant differences were detected among all four groups on day 7 (*p* < 0.001), with the PAW group showing significantly higher levels than the other three groups (Figure 7D). On day 14, IL-10 levels were significantly lower than on day 7 among the three experimental groups, although they remained significantly greater than in the sham group (*p* < 0.001) (Figure 7E).

### 3.6. Oxidative Stress Analysis

A two-way ANOVA was performed to compare MDA and SOD levels in skin tissues across groups on days 7 and 14. The results reveal that the group factor significantly impacted both MDA and SOD levels (MDA: *F* = 15.651, *p* < 0.001; SOD: *F* = 13.23, *p* < 0.001). The Model group exhibited significantly greater MDA levels than the other three groups (*p* < 0.05) (Figure 7C). Compared with the Model and Normal saline groups, the PAW group showed considerably higher SOD levels (*p* < 0.01) (Figure 7F). Overall, PAW treatment was more effective than normal saline irrigation at reducing MDA and increasing SOD.

### 3.7. Biological Safety Analysis

**Figure 8:**
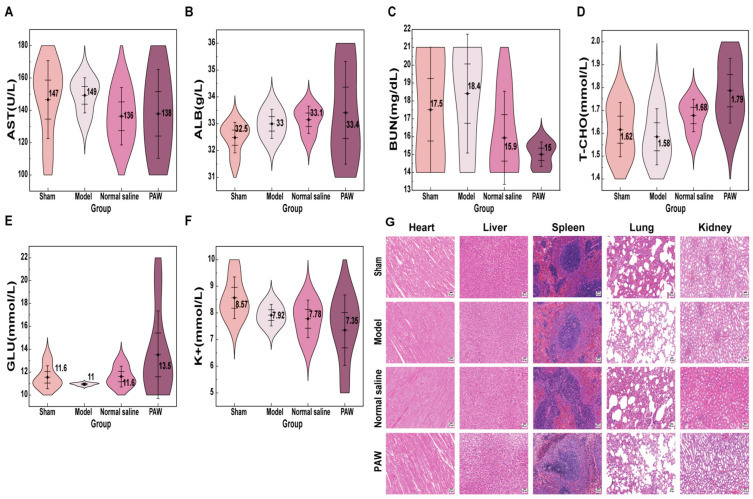
Evaluation of systemic biosafety by serum biochemical indices and histopathology of major organs. (**A**) The concentrations of AST among groups (*n* = 3–5). (**B**) The concentrations of ALB among groups (*n* = 3–5). (**C**) The concentrations of BUN among groups (*n* = 3–5). (**D**) The concentrations of T-CHO among groups (*n* = 3–5). (**E**) The concentrations of GLU among groups (*n* = 3–5). (**F**) The concentrations of K^+^ among groups (*n* = 3–5). (**G**) H&E staining of major organs among groups (*n* = 3–5) (×200), scale bar = 50 µm.

Given that PAW must directly interact with wounds to exert its therapeutic effects, a thorough biosafety evaluation was essential. This study specifically evaluated the safety of its topical application through blood biochemical analysis and histopathological examination of major organs. No significant differences were observed in blood biochemical indices (Figure 8A–F) or visceral tissue structure (Figure 8G) between the Sham, Model, Normal saline, and PAW groups. Overall, no adverse effects on serum biochemistry or major organ histopathology were observed.

## 4. Discussion

**Figure 9:**
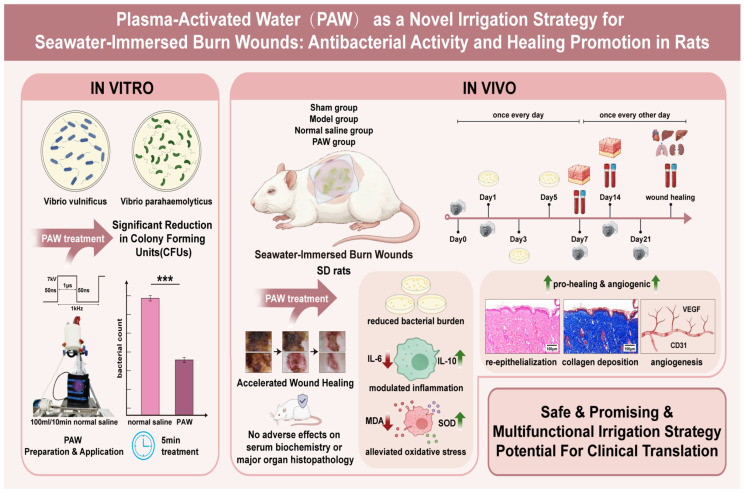
Schematic summary of the results. PAW exhibits strong in vitro antibacterial activity against Vibrio vulnificus and Vibrio parahaemolyticus. In vivo, PAW accelerates wound healing by maintaining antibacterial effects, modulating inflammation, alleviating oxidative stress, and promoting angiogenesis and collagen deposition, with excellent biosafety. *** marked *p* < 0.001. scale bar = 100 µm. Created in BioRender. Liu Tian. (2026). https://BioRender.com/tyg4px6 (accessed on 26 April 2026).

This study demonstrates that PAW significantly improves healing outcomes in seawater-immersed burn wounds. Seawater contamination represents a clinically challenging scenario due to high bacterial load, delayed wound closure, and exaggerated inflammatory responses. Our findings suggest that PAW addresses several of these pathological features simultaneously, including bacterial burden, inflammatory dysregulation, oxidative stress imbalance, and impaired tissue remodeling (Figure 9).

The antibacterial activity of PAW appears to be a critical early determinant of its therapeutic efficacy. It is widely recognized that the bioactivity of PAW arises from the synergistic action of multiple reactive species, particularly ROS and RNS, which, together with an acidic and highly oxidative environment, collectively drive microbial inactivation [23,30,31]. These reactive species induce severe oxidative and nitrosative stress, which directly disrupts bacterial cell membranes, degrades intracellular proteins, and damages microbial DNA, effectively eradicating pathogens and their biofilms without easily inducing bacterial resistance [30,32]. However, the complex and variable composition of the active particles in PAW is influenced by multiple factors, such as plasma discharge methods, excitation voltage, electrode types, and particle-particle reaction probes [33,34]. Furthermore, the limited sensitivity of current detection methods to short-lived reactive intermediates makes it difficult to fully resolve their identities and contributions, and the precise composition and synergistic mechanisms of PAW remain active areas of investigation [35]. Previous studies have identified H_2_O_2_ as the primary ROS and NO_3_^−^ and NO_2_^−^ as the main RNS in PAW. These three long-lived reactive factors are considered crucial for exerting their effects [36]. Therefore, in this study, we characterized the concentrations of the three most significant and long-term active factors in PAW—NO_3_^−^, H_2_O_2_, and NO_2_^−^ using fixed preparation parameters. Based on our preliminary studies, while the bulk concentrations of these long-lived species were largely maintained, a slight but statistically significant temporal decay in H_2_O_2_ and NO_2_^−^ was indeed observed within 120 min at room temperature. Therefore, to circumvent this time-dependent decay of active components and minimize experimental variability, PAW was freshly prepared in all subsequent experiments. Consistent with Thirumdas et al. [23] and Traylor et al. [37], H_2_O_2_ is likely considered to be a fundamental substance in the antibacterial efficacy within PAW, while NO_3_^−^ and NO_2_^−^ prolong the efficacy of PAW’s antibacterial properties. Furthermore, physicochemical analysis revealed that the prepared PAW formed a characteristic acidic and highly oxidative environment, accompanied by increased electrical conductivity. The elevated conductivity reflects the accumulation of dissolved ions and charged reactive species, which interfere with the ion-exchange equilibrium across bacterial cell membranes [23,30,31]. Consistent with the existing literature, the combined physicochemical stress, driven by low pH, high ORP, and high EC, not only directly disrupts the transmembrane proton motive force and osmotic balance but also acts synergistically with ROS/RNS to further compromise membrane permeability, thereby maximizing the overall antibacterial efficacy [23,30,31].

Functionally, our in vitro experiments found significant antibacterial effects of PAW against *Vibrio vulnificus* and *Vibrio parahaemolyticus* within 5 min (*p* < 0.001). Our findings are similar to those of Zhang et al. [32], who reported that PAW quickly killed *Vibrio parahaemolyticus* within seconds. Importantly, compared with normal saline irrigation, PAW significantly decreased the number of viable bacteria in wound exudate (*p* < 0.01), and both methods showed significantly fewer bacteria than the Model group (*p* < 0.05). This suggests that any irrigation treatment is more effective than no watering after standard povidone-iodine disinfection. While the combination of povidone-iodine and normal saline irrigation is a commonly used treatment in clinical practice [13], PAW demonstrates a more effective defense against infection. It maintains its primary cleaning function, and its various ROS and RNS continually target the wound surface to destroy bacteria and inhibit regrowth, creating a cleaner wound environment for subsequent healing.

Wound healing is an active, well-coordinated process that includes hemostasis, inflammation, growth, and reshaping [38]. Among them, the inflammatory phase is vital for initiating repair; however, dysregulated or lingering inflammation may prevent repair and cause tissue damage [39]. IL-6 and IL-10 function as a pair of key inflammatory regulators with opposing roles, and the dynamic equilibrium between them has been crucial for the wound repair process [40]. IL-6 predominantly governs the initial inflammatory phase, enlisting immune cells to eliminate the injury site and initiate repair mechanisms, whereas IL-10 coordinates subsequent anti-inflammatory responses and tissue remodeling to prevent excessive inflammation and promote healing [41]. In accordance with previous studies [24,42], on day 1, PAW immediately promoted the release of Interleukin-1β (IL-1)β, IL-6, and IL-10, then reduced them from days 3 to 5, shortening the inflammatory response. In this study, on days 7 and 14, PAW was shown to effectively modulate the inflammatory response in seawater-immersed burn wounds, as evidenced by a significant decrease in IL-6 and an increase in IL-10 (*p* < 0.05). Importantly, HE staining demonstrated that the effect of PAW was time-dependent. On day 7, Model group exhibited markedly greater inflammatory cell infiltration than all other groups (*p* < 0.001), reflecting sustained and excessive inflammation under seawater-contaminated conditions. On day 14, the inflammatory cell infiltration area in PAW group was significantly lower than in both the Model and Normal saline groups (*p* < 0.05), suggesting more efficient resolution of inflammation. These findings are consistent with the cytokine results, suggesting that PAW not only orchestrates a transient, controlled inflammatory response but also accelerates its timely resolution, thereby preventing prolonged inflammatory injury. Consequently, the wound microenvironment quickly shifted from a pro-inflammatory state to a tissue-repair state.

Furthermore, oxidative stress is a well-established barrier to healing [43]. Oxidative stress has an important function in wound healing. The relative levels of MDA to SOD provide a key indicator of oxidative stress, directly influencing cellular events critical to wound repair [44]. Our findings demonstrated that seawater-immersed burn wounds disrupted redox homeostasis and exacerbated oxidative stress (*p* < 0.001), as reported in a prior study [45]. Additionally, PAW significantly reduced oxidative damage, as evidenced by lower MDA levels and higher SOD activity (*p* < 0.01). We believe that the efficacy of PAW might be attributed to active substances such as ROS and RNS. After contact with the wound, these species might indirectly alleviate oxidative damage through antibacterial effects and the regulation of inflammatory pathways [46,47]. Furthermore, at suitable concentrations, these molecules might function as redox signaling agents, activating endogenous antioxidant defenses by inducing mild and manageable oxidative stress, ultimately enhancing the body’s capacity to resolve inflammation and counteract oxidative injury [43,48,49].

Our histological analyses further substantiated the therapeutic advantage of PAW. Masson staining demonstrated significant group and time effects on collagen volume fraction (CVF) (*p* < 0.001). All experimental groups showed increased collagen deposition from day 7 to day 14 (*p* < 0.01), reflecting the transition from inflammation to the proliferative and remodeling phases. Notably, CVF values followed the order PAW group > Normal saline group > Model group (*p* < 0.01), indicating that PAW significantly enhanced collagen synthesis and maturation. Moreover, collagen fibers in PAW group appeared more organized and mature. These findings suggest that PAW not only accelerates wound closure but also improves the structural quality and integrity of the regenerated tissue. Furthermore, although macroscopic measurements of wound area and healing rates between the PAW and Normal saline groups did not reach statistical significance, this may be primarily due to inherent individual variability and the lower statistical power resulting from the small sample size. Nevertheless, the consistently higher trends in the PAW group, combined with these significant histological improvements, suggest the potential biological advantages of PAW in promoting wound repair.

Angiogenesis is a crucial step in wound repair and healing, which is essential for delivering oxygen and nutrients to regenerated tissue [50]. VEGF and CD31 have been identified as factors associated with angiogenesis that can stimulate cell differentiation and proliferation, serving as markers of angiogenesis [40,51]. In this study, the coordinated upregulation of VEGF and a parallel increase in MVD indicated that PAW positively stimulated vascularization at the wound site (*p* < 0.01). Adequate vascularization is closely linked to controlled inflammation and a balanced oxidative status [43]. We hypothesize that ROS within the PAW may act as redox signaling molecules capable of stabilizing or activating multiple transcription regulators, notably nuclear element-KB (NF-KB) and activation protein-1 (AP-1), with particular emphasis on hypoxia-inducible factor-1 alpha (HIF-1α), starting the scripting for downstream target proteins, most notably VEGF [43,52]. On the other hand, RNS within the PAW might cooperate with VEGF to increase local blood flow and oxygen delivery, thereby promoting endothelial cell proliferation and migration [43,53]. In the end, this behavior significantly increases the number of CD31-positive microvessels, promoting angiogenesis and advancing wound healing [54,55]. Nevertheless, the specific role of PAW in activating this pathway is not yet proven; confirming its involvement is a critical next step.

Safety is a prerequisite for clinical translation. Other studies that performed biosafety experiments in mouse models with skin trauma reported that PAW had no substantial influence on the external appearance, structure, tissue architecture, cellular composition, or active serum biological variables of the mice [20,56]. In this study, our biosafety evaluation focused on comparing histological features of the viscera and major blood biochemical parameters in rats. Although PAW is administered topically, its long-lived reactive species may potentially enter systemic circulation, and excessive ROS/RNS are known to contribute to tissue injury and organ dysfunction [57,58]. Therefore, biochemical markers, including AST, ALB, and BUN, which are widely recognized indicators of hepatic and renal function, together with T-CHO, GLU, and K^+^, which reflect metabolic and electrolyte homeostasis, were monitored to evaluate potential systemic effects [59,60]. No differences in histological structure were noted in the visceral organs of rats. Furthermore, no significant differences in biochemical markers were observed across the groups, indicating that topical PAW does not disrupt organ function or systemic metabolic balance. Consequently, these findings determine that PAW is safe for administration in rats.

Beyond safety and efficacy, the clinical translation of PAW also depends on its economic viability, clinical scalability, and regulatory framework. Economically, early plasma generation relied on bulky, expensive laboratory setups, but several recent studies have developed energy-efficient, portable devices capable of on-site, on-demand PAW preparation [24,56,61]. The economic feasibility of PAW technology has been preliminarily validated in the agri-food sector, where the process cost of PAW treatment in agricultural product processing has been reported to be less than $0.5 per ton [62]. Although the initial capital investment in equipment acquisition and underlying research and development represents considerable upfront expenditure, PAW synthesis requires only inexpensive consumables such as saline solution and electricity, enabling the simultaneous generation of a diverse array of reactive species that promote tissue repair through multiple targets, thereby offering a green and sustainable option for long-term wound care [24,30,56]. Furthermore, while our study exclusively focuses on the application of PAW during the initial irrigation phase of wound management, recent research has explored formulating PAW into various topical dosage forms [63] or combining it with existing therapeutic dressings [64] to overcome the rapid degradation of reactive species and further expand clinical applicability. Biocompatible hydrogel matrices, for instance, can utilize three-dimensional networks to stabilize active species and achieve sustained release [65]. In theory, a single preparation device could be used for both initial wound irrigation and the preparation of various topical PAW formulations, enabling multi-modal, phased, and comprehensive wound management. This approach might have the potential for cost-effectiveness and clinical scalability; however, there is currently insufficient research, and it still requires systematic evaluation and validation. Cost-effectiveness and scalable system design, including the development of affordable, portable plasma devices, might be essential to fully realize the clinical translation of PAW [66].

More importantly, a critical determinant of clinical scalability is the establishment and implementation of standardized treatment protocols [63]. In our study, the irrigation protocol was determined based on in vitro results and prior wound care experience; we did not conduct systematic investigations into dosing parameters and administration frequency, which remain to be further optimized. Furthermore, from a regulatory perspective, beyond parameter optimization, clinical translation of PAW therapy also faces challenges in long-term efficacy and safety evaluation, mechanistic elucidation, preparation stability and controllability, and measurement and storage stability of the active species [67,68]. The regulatory framework for plasma medicine remains nascent, lacking unified standards for physicochemical characterization, biomedical effect assessment, and clinical trial design, thereby hindering comparability, reproducibility, and approval [67]. Although certain CAP devices have obtained Conformité Européenne marking as Class IIa/IIb medical devices in the European Union, offering a reference pathway [68], the regulatory classification of PAW remains unresolved. Advancing PAW translation thus requires clarifying mechanisms, establishing standardized dose-effect systems, unifying quantification methods for active species, improving clinical trial practices, and promoting harmonization of international standards.

## 5. Conclusions

This study demonstrates that PAW significantly promotes the healing of seawater-immersed burn wounds in male SD rats. We made PAW by activating normal saline with a transportable plasma device. An in vitro study confirmed the effective inactivation of *Vibrio vulnificus* and *Vibrio parahaemolyticus* by PAW under controlled conditions. Animal studies have shown that PAW significantly improved the healing of seawater-immersed burn wounds, likely by coordinating control of infection, inflammation, oxidative stress, and tissue remodeling. The rapid antibacterial effects can promptly alleviate inflammation and oxidative damage, then enhance granulation, collagen deposition, and angiogenesis to achieve more efficient, high-quality, and safe wound recovery. PAW demonstrates significant potential as a multipurpose, ready-to-use irrigation solution for clinical treatments.

However, this study has several limitations. First, the stability of PAW was not thoroughly assessed, and the characterization of its active particles remains limited; due to their rapid degradation and the limited sensitivity of current detection methods, short-lived reactive intermediates were not fully profiled. Second, despite standardized modeling protocols, individual biological differences and a relatively small sample size may have introduced inherent heterogeneity in final burn wound depths, potentially limiting the statistical power of macroscopic measurements. Furthermore, while the exclusive inclusion of male rats was necessary to maintain baseline consistency, it restricted the generalizability of our findings across sexes. Third, the current PAW irrigation regimen was empirically designed based on in vitro results and general wound healing experience, lacking a systematic optimization of dose–response relationships and intervention frequency. Fourth, although laboratory assays were conducted in a blinded manner, macroscopic wound evaluations and data analyses were performed unblinded, which may have potentially introduced observer bias. Future studies should first fully identify the active components in PAW and delve deeper into the specific mechanisms underlying their healing-promoting effects. Additionally, subsequent research should increase sample size, include female subjects, and systematically evaluate the effects of different dosages and administration frequencies to optimize the topical treatment regimen.

## Figures and Tables

**Table 1:** Physicochemical properties of PAW compared with control.

Parameter	Control	PAW (*n* = 3, $\bar{x}$ ± s)
pH	4.58	3.24 ± 0.03
ORP (mV)	98	175.7 ± 1.2
EC (mS/cm)	14.87	15.13 ± 0.02

## Data Availability

The data analyzed during the current study are available from the corresponding authors on request.
